# Supplementary material for: Characterizing the cognitive and mental health benefits of exercise and video game playing
Source: PLoS One. 2025 Oct 24;20(10):e0334924. doi: 10.1371/journal.pone.0334924 (PMC12551924; doi:10.1371/journal.pone.0334924)
Supplement: S1 Table — (DOCX) [file pone.0334924.s001.docx]

**S1 Table: Descriptive Statistics of all Cognitive Tests.**

|  | **count** | **mean** | **std** | **min** | **25%** | **50%** | **75%** | **max** |
| --- | --- | --- | --- | --- | --- | --- | --- | --- |
| SS_max_score | 923 | 5.51 | 1.04 | 2 | 5 | 5 | 6 | 9 |
| GR_final_score | 923 | 17.75 | 5.02 | 1 | 15 | 18 | 21 | 33 |
| DT_final_score | 923 | 21.35 | 15.63 | -19 | 7 | 24 | 34 | 65 |
| OOO_max | 923 | 15.58 | 2.12 | 5 | 14 | 16 | 17 | 20 |
| ML_max_score | 923 | 7.38 | 1.12 | 3 | 7 | 7 | 8 | 13 |
| RT_final_score | 923 | 75.62 | 34.49 | -23 | 52 | 76 | 98 | 194 |
| FM_final_score | 923 | 108.80 | 30.10 | 9 | 90 | 110 | 128 | 210 |
| DS_max_score | 923 | 6.86 | 1.42 | 4 | 6 | 7 | 8 | 14 |
| SP_final_score | 923 | 19.32 | 8.23 | 2 | 14 | 18 | 24 | 52 |
| PA_max_score | 923 | 4.51 | 0.95 | 2 | 4 | 4 | 5 | 8 |
| PO_final_score | 923 | 38.20 | 20.52 | -11 | 23 | 36 | 52 | 111 |
| TS_max_score | 923 | 7.93 | 1.62 | 4 | 7 | 8 | 9 | 14 |
