# Supplementary material for: Characterizing the cognitive and mental health benefits of exercise and video game playing
Source: PLoS One. 2025 Oct 24;20(10):e0334924. doi: 10.1371/journal.pone.0334924 (PMC12551924; doi:10.1371/journal.pone.0334924)
Supplement: S2 Table — (DOCX) [file pone.0334924.s002.docx]

**S2 Table. Results of Likelihood Ratio Tests for Lifestyle Factors Across all Brain Health Scores.**

| **contrast** | **score** | **df.h1** | **LL.h1** | **df.LR** | **LR** | **p.unc** | **p.adj** |
| --- | --- | --- | --- | --- | --- | --- | --- |
| gamer_type | STM | 8 | -1213.22 | 2 | 18.68 | < 0.001 | 0.001 |
| gamer_type | reasoning | 8 | -1195.75 | 2 | 21.78 | < 0.001 | < 0.001 |
| gamer_type | verbal | 8 | -1284.20 | 2 | 2.43 | 0.297 | 1.000 |
| gamer_type | processing_speed | 8 | -1111.75 | 2 | 24.06 | < 0.001 | < 0.001 |
| gamer_type | overall | 8 | -1093.20 | 2 | 41.61 | < 0.001 | < 0.001 |
| gamer_type | phq2 | 13 | -894.65 | 2 | 7.20 | 0.027 | 0.383 |
| gamer_type | gad2 | 10 | -552.36 | 2 | 0.16 | 0.922 | 1.000 |
| passed_who_guidelines | STM | 8 | -1213.22 | 1 | 2.66 | 0.103 | 1.000 |
| passed_who_guidelines | reasoning | 8 | -1195.75 | 1 | 3.87 | 0.049 | 0.687 |
| passed_who_guidelines | verbal | 8 | -1284.20 | 1 | 0.02 | 0.901 | 1.000 |
| passed_who_guidelines | processing_speed | 8 | -1111.75 | 1 | 0.99 | 0.320 | 1.000 |
| passed_who_guidelines | overall | 8 | -1093.20 | 1 | 0.12 | 0.730 | 1.000 |
| passed_who_guidelines | phq2 | 13 | -894.65 | 1 | 12.37 | < 0.001 | 0.006 |
| passed_who_guidelines | gad2 | 10 | -552.36 | 1 | 9.29 | 0.002 | 0.032 |
