# Supplementary material for: Characterizing the cognitive and mental health benefits of exercise and video game playing
Source: PLoS One. 2025 Oct 24;20(10):e0334924. doi: 10.1371/journal.pone.0334924 (PMC12551924; doi:10.1371/journal.pone.0334924)
Supplement: S3 Table — Pairwise differences in the marginal predicted probabilities of mental health scores between levels of physical activity (yes – no; i.e., met WHO guidelines vs did not meet WHO guidelines). p-values were Bonferroni corrected for N = 6 comparison for PHQ-2 and N = 4 for the GAD-2. SE = standard error, z.ratio = z-statistics, p.adj = corrected p-value. (DOCX) [file pone.0334924.s003.docx]

**S3 Table. Pairwise Differences in Marginal Predicted Probabilities of Mental Health Scores.** Pairwise differences in the marginal predicted probabilities of mental health scores between levels of physical activity (yes - no; i.e., met WHO guidelines vs did not meet WHO guidelines). *p*-values were Bonferroni corrected for N=6 comparison for PHQ-2 and N=4 for the GAD-2. *SE = standard error, z.ratio = z-statistics, p.adj = corrected p-value.*

| **scale** | **score** | **contrast** | **estimate** | **SE** | **z.ratio** | **p.adj** |
| --- | --- | --- | --- | --- | --- | --- |
| PHQ2 | 0 | yes - no | 0.12 | 0.03 | 3.50 | 0.003 |
| PHQ2 | 1 | yes - no | -0.02 | 0.01 | -3.33 | 0.006 |
| PHQ2 | 2 | yes - no | -0.05 | 0.02 | -3.39 | 0.005 |
| PHQ2 | 3 | yes - no | -0.01 | 0.01 | -2.72 | 0.045 |
| PHQ2 | 4 | yes - no | -0.01 | 0.00 | -2.45 | 0.101 |
| PHQ2 | 5 | yes - no | -0.01 | 0.00 | -2.19 | 0.198 |
| PHQ2 | 6 | yes - no | -0.01 | 0.01 | -2.51 | 0.086 |
| GAD2 | 0-1 | yes - no | 0.10 | 0.03 | 2.96 | 0.012 |
| GAD2 | 2-3 | yes - no | -0.06 | 0.02 | -2.99 | 0.011 |
| GAD2 | 4-5 | yes - no | -0.02 | 0.01 | -2.58 | 0.040 |
| GAD2 | 6 | yes - no | -0.01 | 0.01 | -2.25 | 0.098 |
