# Supplementary material for: Characterizing the cognitive and mental health benefits of exercise and video game playing
Source: PLoS One. 2025 Oct 24;20(10):e0334924. doi: 10.1371/journal.pone.0334924 (PMC12551924; doi:10.1371/journal.pone.0334924)
Supplement: S4 Table — Results for all pairwise tests of differences in the marginal means between gaming subtypes, within each cognitive score. p-values were Bonferroni corrected for N = 3 comparisons. SE = standard error, df = degrees of freedom, t.stat = t-statistic, p.adj = corrected p-value. (DOCX) [file pone.0334924.s004.docx]

**S4 Table. Pairwise Tests of Differences in Marginal Means Between Gaming Subtypes.** Results for all pairwise tests of differences in the marginal means between gaming subtypes, within each cognitive score. *p*-values were Bonferroni corrected for N=3 comparisons. *SE = standard error, df = degrees of freedom, t.stat = t-statistic, p.adj = corrected p-value.*

| **cog.score** | **contrast** | **estimate** | **SE** | **df** | **t.stat** | **p.adj** |
| --- | --- | --- | --- | --- | --- | --- |
| STM | Infrequent_Gamer - Non_Gamer | 0.20 | 0.07 | 914 | 2.87 | 0.012 |
| STM | Frequent_Gamer - Non_Gamer | 0.36 | 0.09 | 914 | 4.23 | < 0.001 |
| STM | Frequent_Gamer - Infrequent_Gamer | 0.16 | 0.08 | 914 | 2.01 | 0.134 |
| reasoning | Infrequent_Gamer - Non_Gamer | 0.14 | 0.07 | 914 | 2.01 | 0.133 |
| reasoning | Frequent_Gamer - Non_Gamer | 0.39 | 0.08 | 914 | 4.66 | < 0.001 |
| reasoning | Frequent_Gamer - Infrequent_Gamer | 0.25 | 0.08 | 914 | 3.23 | 0.004 |
| verbal | Infrequent_Gamer - Non_Gamer | -0.07 | 0.08 | 914 | -0.89 | 1.000 |
| verbal | Frequent_Gamer - Non_Gamer | 0.06 | 0.09 | 914 | 0.66 | 1.000 |
| verbal | Frequent_Gamer - Infrequent_Gamer | 0.13 | 0.09 | 914 | 1.50 | 0.399 |
| overall | Infrequent_Gamer - Non_Gamer | 0.19 | 0.06 | 914 | 3.04 | 0.007 |
| overall | Frequent_Gamer - Non_Gamer | 0.48 | 0.07 | 914 | 6.49 | < 0.001 |
| overall | Frequent_Gamer - Infrequent_Gamer | 0.30 | 0.07 | 914 | 4.30 | < 0.001 |
| p.speed | Infrequent_Gamer - Non_Gamer | 0.19 | 0.06 | 914 | 2.99 | 0.009 |
| p.speed | Frequent_Gamer - Non_Gamer | 0.37 | 0.08 | 914 | 4.87 | < 0.001 |
| p.speed | Frequent_Gamer - Infrequent_Gamer | 0.18 | 0.07 | 914 | 2.59 | 0.029 |
