# Supplementary material for: Characterizing the cognitive and mental health benefits of exercise and video game playing
Source: PLoS One. 2025 Oct 24;20(10):e0334924. doi: 10.1371/journal.pone.0334924 (PMC12551924; doi:10.1371/journal.pone.0334924)
Supplement: S5 Table — H1 = degrees of freedom for the full (H1) model, LL.H1 = Log Likelihood of the full model, df = degrees of freedom difference between full and nested (H0) models (also the df of the Chisq test), LR = likelihood ratio, p.unc = uncorrected p-value, p.adj = corrected p-value. (DOCX) [file pone.0334924.s005.docx]

**S5 Table. Results of Likelihood Ratio Tests for Cognitive Score Testing Interactions Between Age and Lifestyle Factors.** *df.h1 = degrees of freedom for the full (H1) model, LL.h1 = Log Likelihood of the full model, df = degrees of freedom difference between full and nested (H0) models (also the df of the Chisq test), LR = likelihood ratio, p.unc = uncorrected p-value, p.adj = corrected p-value.*

| **contrast** | **score** | **df.h1** | **LL.h1** | **df** | **LR** | **p.unc** | **p.adj** |
| --- | --- | --- | --- | --- | --- | --- | --- |
| age_by_passed_who_guidelines | GAD2 | 12 | -308.41 | 1 | 2.55 | 0.110 | 1.000 |
| age_by_passed_who_guidelines | PHQ2 | 15 | -543.64 | 1 | 0.00 | 0.976 | 1.000 |
| age_by_passed_who_guidelines | STM | 11 | -830.45 | 1 | 1.97 | 0.161 | 1.000 |
| age_by_passed_who_guidelines | reasoning | 11 | -805.43 | 1 | 1.18 | 0.277 | 1.000 |
| age_by_passed_who_guidelines | verbal | 11 | -898.47 | 1 | 0.00 | 0.945 | 1.000 |
| age_by_passed_who_guidelines | overall | 11 | -739.85 | 1 | 0.07 | 0.797 | 1.000 |
| age_by_passed_who_guidelines | p.speed | 11 | -805.68 | 1 | 3.29 | 0.070 | 0.975 |
| age_by_gamer_type | GAD2 | 13 | -307.75 | 2 | 3.86 | 0.145 | 1.000 |
| age_by_gamer_type | PHQ2 | 16 | -543.60 | 2 | 0.08 | 0.962 | 1.000 |
| age_by_gamer_type | STM | 12 | -830.04 | 2 | 2.79 | 0.247 | 1.000 |
| age_by_gamer_type | reasoning | 12 | -805.97 | 2 | 0.10 | 0.950 | 1.000 |
| age_by_gamer_type | verbal | 12 | -898.46 | 2 | 0.02 | 0.988 | 1.000 |
| age_by_gamer_type | overall | 12 | -738.99 | 2 | 1.78 | 0.410 | 1.000 |
| age_by_gamer_type | p.speed | 12 | -806.09 | 2 | 2.46 | 0.292 | 1.000 |
