# Supplementary material for: Characterizing the cognitive and mental health benefits of exercise and video game playing
Source: PLoS One. 2025 Oct 24;20(10):e0334924. doi: 10.1371/journal.pone.0334924 (PMC12551924; doi:10.1371/journal.pone.0334924)
Supplement: Attachment — Submitted filename: Response to Reviewers.docx [file pone.0334924.s007.docx]

**Response to Reviewers**

PONE-D-25-17797

Characterizing the Cognitive and Mental Health Benefits of Exercise and Video Game Playing

PLOS ONE

Dr. Mohammad Mofatteh, PhD, MPH, MSc, PGCert, BSc (Hons), MB BCh (c)
Academic Editor
PLOS ONE

Dear Dr. Mofatteh,

Thank you very much for your response, for the Reviewers’ thorough assessment of our manuscript, and for allowing us the opportunity to incorporate all necessary revisions. We greatly appreciate your consideration, and we have addressed all of the points raised below with our responses in blue font.

Overall, aside from formatting revisions, we noted that the Reviewers’ comments primarily focused on certain limitations related to our study design and measures. We agree with all these points, and within the manuscript we have acknowledged these limitations and how they might affect our results. We believe that these weaknesses, common to many such cross-sectional studies, are offset somewhat by the advantages of our design; specifically, the ability to collect a rich dataset (i.e., multiple standardized questionnaires and a comprehensive cognitive battery) from a large and diverse sample of the population. It is also worth noting that the Reviewers did not raise any concerns, statistical or otherwise, about how we analyzed this comprehensive dataset to test our hypotheses.

Regardless, we maintain that our study’s limitations do not discount or undermine the importance of our findings. Among a large sample of adults with a tendency toward regular exercise, we observed a positive relationship between self-reported exercise levels and mental health but not cognition, and a positive relationship between video gaming and cognition but not mental health. As we state in the manuscript: “*Despite the limitations noted here, the fact that we observed some significant associations for both cognitive and mental health, and exercise and video-gaming, suggests that the null results are not simply due to inadequacies of the instruments used, nor an insufficient sample size.*” Nevertheless, on the basis of the Reviewers’ feedback, we have elaborated on our methodological decisions to provide readers with additional clarification. We have also justified the use of certain measures as they relate to the present study design.

Thank you again for reconsidering our manuscript. Should anything remain unclear or if there are any additional points requiring our attention, please do not hesitate to contact us.

Sincerely,

Sydni Paleczny ([spaleczn@uwo.ca](mailto:spaleczn@uwo.ca)) & Conor Wild ([cwild@uwo.ca](mailto:cwild@uwo.ca)) on behalf of all co-authors.

Journal Requirements:

When submitting your revision, we need you to address these additional requirements.

1. Please ensure that your manuscript meets PLOS ONE's style requirements, including those for file naming. The PLOS ONE style templates can be found at <https://journals.plos.org/plosone/s/file?id=wjVg/PLOSOne_formatting_sample_main_body.pdf> and <https://journals.plos.org/plosone/s/file?id=ba62/PLOSOne_formatting_sample_title_authors_affiliations.pdf>

**1. Response from authors:** Thank you for pointing us to the correct guidelines. All formatting has now been updated within the main manuscript, title page, and supporting information to adhere to these requirements.

2. Thank you for stating the following in the Competing Interests section:
The cognitive tests used in this study are marketed by Creyos (formerly Cambridge Brain Sciences), of which A.M.O. is the chief scientific officer, C.J.W. is the director of data science, and S.G.P. is staff scientist. Under the terms of the existing licensing agreement, A.M.O. and his collaborators are free to use the platform at no cost for their scientific studies, and that such research projects neither contribute to, nor are influenced by, the activities of the company. Consequently, there is no overlap between the present study and the activities of Creyos, nor was there any cost to the authors, funding bodies, or participants who were involved in the study.

Please confirm that this does not alter your adherence to all PLOS ONE policies on sharing data and materials, by including the following statement: "This does not alter our adherence to PLOS ONE policies on sharing data and materials.” (as detailed online in our guide for authors <http://journals.plos.org/plosone/s/competing-interests>). If there are restrictions on sharing of data and/or materials, please state these. Please note that we cannot proceed with consideration of your article until this information has been declared.

Please include your updated Competing Interests statement in your cover letter; we will change the online submission form on your behalf.

**2. Response from authors:** We confirm that our declaration of competing interests does not alter our adherence to all PLOS ONE policies, and we have added the required statement to our Cover Letter declaration as follows:

*“The cognitive tests used in this study are marketed by Creyos (formerly Cambridge Brain Sciences), of which A.M.O. is the chief scientific officer, C.J.W. is the director of data science, and S.G.P. is staff scientist. Under the terms of the existing licensing agreement, A.M.O. and his collaborators are free to use the platform at no cost for their scientific studies, and that such research projects neither contribute to, nor are influenced by, the activities of the company. Consequently, there is no overlap between the present study and the activities of Creyos, nor was there any cost to the authors, funding bodies, or participants who were involved in the study.* ***This does not alter our adherence to PLOS ONE policies on sharing data and materials.”***

Thank you for updating this within the online submission form on our behalf.

3. [Please remove all personal information, ensure that the data shared are in accordance with participant consent, and re-upload a fully anonymized data set.

Note: spreadsheet columns with personal information must be removed and not hidden as all hidden columns will appear in the published file.

Additional guidance on preparing raw data for publication can be found in our Data Policy (https://journals.plos.org/plosone/s/data-availability#loc-human-research-participant-data-and-other-sensitive-data) and in the following article: http://www.bmj.com/content/340/bmj.c181.long.](http://track.editorialmanager.com/CL0/http:%2F%2Fwww.bmj.com%2Fcontent%2F340%2Fbmj.c181.long./1/010f0196e65e3044-7efc2bc1-d65e-4b80-9414-44b41640679a-000000/FmyldLAHZoIJk9uw1hJcTDX3inmQ8zRoarTGZycnDV0=211)

**3. Response from authors:** The complete raw dataset, including individual raw files and a final pre-processed dataset as described in the manuscript, are available at <https://borealisdata.ca/dataset.xhtml?persistentId=doi:10.5683/SP3/WUYAGU>. Participants consented for their fully anonymized data to be made available on this institutional dataverse repository. All identifying information has been removed from the individual data and preprocessed files.

4. [Please include captions for your Supporting Information files at the end of your manuscript, and update any in-text citations to match accordingly. Please see our Supporting Information guidelines for more information: http://journals.plos.org/plosone/s/supporting-information.](http://track.editorialmanager.com/CL0/http:%2F%2Fjournals.plos.org%2Fplosone%2Fs%2Fsupporting-information./1/010f0196e65e3044-7efc2bc1-d65e-4b80-9414-44b41640679a-000000/vqEOXvuFBqkKZATiEiOtMKJwstnhO2CzTpd3zu_qvB0=211)

**4. Response from authors:** Thank you for directing us to the corresponding formatting requirements. We have updated all in-text citations (e.g., “S1 Fig”) and added “Supporting information” as a Level 1 heading at the bottom of the manuscript which lists all file titles and labels.

Additional Editor Comments:

Please provide a point-by-point and detailed response to all the reviewers' comments.


Reviewers' comments:

Reviewer's Responses to Questions

**Comments to the Author**

1. Is the manuscript technically sound, and do the data support the conclusions?

The manuscript must describe a technically sound piece of scientific research with data that supports the conclusions. Experiments must have been conducted rigorously, with appropriate controls, replication, and sample sizes. The conclusions must be drawn appropriately based on the data presented.

Reviewer #1: Partly

Reviewer #2: Yes

2. Has the statistical analysis been performed appropriately and rigorously?

Reviewer #1: Yes

Reviewer #2: Yes

3. Have the authors made all data underlying the findings in their manuscript fully available?

The [PLOS Data policy](http://track.editorialmanager.com/CL0/http:%2F%2Fwww.plosone.org%2Fstatic%2Fpolicies.action%23sharing/1/010f0196e65e3044-7efc2bc1-d65e-4b80-9414-44b41640679a-000000/_ab1xx9xVDCNEC_LGfkochp7pAk3RZX98JT7nPsNTNk=211) requires authors to make all data underlying the findings described in their manuscript fully available without restriction, with rare exception (please refer to the Data Availability Statement in the manuscript PDF file). The data should be provided as part of the manuscript or its supporting information, or deposited to a public repository. For example, in addition to summary statistics, the data points behind means, medians and variance measures should be available. If there are restrictions on publicly sharing data—e.g. participant privacy or use of data from a third party—those must be specified.

Reviewer #1: Yes

Reviewer #2: Yes

4. Is the manuscript presented in an intelligible fashion and written in standard English?

PLOS ONE does not copyedit accepted manuscripts, so the language in submitted articles must be clear, correct, and unambiguous. Any typographical or grammatical errors should be corrected at revision, so please note any specific errors here.

Reviewer #1: Yes

Reviewer #2: Yes

5. Review Comments to the Author

Please use the space provided to explain your answers to the questions above. You may also include additional comments for the author, including concerns about dual publication, research ethics, or publication ethics. (Please upload your review as an attachment if it exceeds 20,000 characters)

Reviewer #1: 1. Minor formatting issues should be addressed—for example, in the Methods section, some subsection headers appear at the bottom of a page with text continuing on the next, which affects readability.

**1. Response from authors:** Thank you for bringing this to our attention. Originally, this issue may have been due to file type conversions – our apologies. We have updated the formatting accordingly so that all section and subsection headers appear directly above the following text. We have also updated all formatting requirements as per the [PLOS guidelines](https://journals.plos.org/plosone/s/file?id=wjVg/PLOSOne_formatting_sample_main_body.pdf).

2. In the Discussion, the authors justify using categorical self-report data over a continuous measure for classifying gaming frequency. A brief explanation of why this approach is preferable would improve clarity.

**2. Response from authors:** Thank you. We agree that further clarification of this method would be helpful for readers. We have therefore elaborated on why this categorical approach was used for classifying gaming frequency in the Methods section *(clean Manuscript Page 6, Line 165; tracked changes Manuscript Page 6 Line 336):*

*“Previous studies have shown that, where self-report data is concerned, the categorization of individuals into extremes based on their reported quantity or frequency of the behaviour (e.g., whether or not they met activity guidelines, based on self-reported minutes) is more reliable than the continuous reported measure (36,53). For example, in their original publication of the PAAQ, Colley et al. show that despite weak correlations (best case Pearson r=0.36) between self-reported physical activity and objective measurements from accelerometers, the proportion of participants that met physical activity guidelines according to these methods were somewhat similar (39% vs. 46%) and these classifications were in agreement 67% of the time. Similarly, Bediou et al. (32) note that the video game questionnaire used in this study was designed only to distinguish frequent gamers from those who partake very infrequently.”*

3. There are some limitations that should be adequately addressed:

a) Cross-sectional design: Causality cannot be established (e.g., do healthier individuals exercise more, or does exercise improve health?). The authors acknowledge this and suggest future longitudinal studies.

**3. a) Response from authors:** Thank you for raising these important points. We agree that this is a limitation that must be addressed, and have added additional detail to the following paragraph of our Discussion section with **additions in bold** (*clean Manuscript Page 27, Line 600; tracked changes Manuscript Page 27 Line 866)*:

**“***While we observed significant associations between physical exercise and mental health and between video gaming and cognitive performance, it is not possible to say that this is a causative relationship based on the current data. For example, more depressed or more anxious individuals may be less likely to exercise than their less depressed or less anxious counterparts. This explanation seems unlikely, however, because the association between physical activity and mental health scores was weakest among those with higher depression or anxiety ratings, suggesting that these symptoms were not the main drivers of the performance differences. Where video gaming and cognition is concerned, the direction of the effect seems rather less open to question. That is to say, while it is theoretically possible that those who have better cognition are more likely to play video games regularly, it seems more probable that regular gaming exerts a direct effect on aspects of cognitive performance*. ***In any case, the cross-sectional design of the present study does not allow us to draw any definitive conclusions surrounding causality. Future research may benefit from longitudinal follow-up to further investigate and determine the nature of this relationship over time****.”*

b) Self-report bias: Physical activity and gaming hours were self-reported. While categorization may reduce inaccuracies, objective measures (e.g., accelerometers) would strengthen the validity of the findings.

**3. b) Response from authors:** Thank you for this suggestion. We agree that it’s worth discussing the use of objective measures (and the challenges with this approach in relation to our methodological design) in order to build upon findings and improve future research. We have therefore added the following detail to the Discussion section with **additions in bold** (*clean Manuscript Page 26, Line 561; tracked changes Manuscript Page 26 Line 822)*:

*“We also must acknowledge the limitations of using self-reported estimates of the frequency and duration of physical exercise and playing video games* ***as it is well known that people over-report*** *(51,101). To overcome this, we categorized individuals based on their self-reported frequency of the activity; although the accuracy of individuals’ self-report judgements may be low, the classification of individuals into categories (i.e., high or low extremes) based on their estimates is substantially better (37,52,53).* ***Obviously, objective data would produce more accurate measurements, even for classifying individuals (102), but within the present study obtaining gold standard accelerometer data was simply not feasible due to the international online design and costs associated with our large sample size.*** ***Personal activity monitors (e.g., FitBit, Apple Watch, etc.) provide an interesting potential alternative for acquiring objective physical activity data, but there are significant issues that first need to be overcome, such as addressing concerns related to data privacy, establishing the quality and reliability of data collected from dozens of different device types across multiple platforms with proprietary measurements, and the need for techniques to harmonize data from across these platforms (102). Future work will investigate the feasibility of incorporating participants’ existing personal physical activity tracking devices into an online study of this type.***

c) Mental health measures: The GAD-2 and PHQ-2 are brief screening tools, not comprehensive diagnostic instruments. As such, the study may overlook more nuanced mental health effects.

**3. c) Response from authors:** Thank you for raising this important point. We have considered and acknowledged the limitations of using brief screening tools, although we feel they ultimately do not discount the importance of the present findings. For example, one could argue that the limited mental health screeners might produce null results, but we have instead shown that associations between (self-reported) physical activity and mental health *can* be detected with these simple two-item questionnaires. Furthermore, our decision to use these screeners was informed by our previous research (Wild et al., 2022) in which we demonstrated an association between mental health (assessed with these screeners), demographics, and long-term symptoms of COVID-19 infection.

Nevertheless, we fully agree that a more complete picture of mental health provided by more comprehensive instruments could reveal more nuanced effects related to mental health. For additional clarity, we have specified “GAD-2 and PHQ-2” within this section of the Discussion (*clean Manuscript Page 25, Line 552; tracked changes Manuscript Page 25 Line 813)*:

“*First,* *there are inadequacies in the instruments used to measure our independent and dependent variables*.” Specifically, “*mental health outcomes were captured using brief psychological screeners* ***(GAD-2 and PHQ-2)*** *intended to identify individuals at risk of anxiety and depression. It is important to acknowledge that these two markers alone provide an incomplete picture of mental health, and that exercise and video-gaming may be associated with improvements in other aspects of mental well-being*.”

d) Sample demographics: The sample is skewed toward older adults (mean age 55) with high exercise adherence (73% met WHO guidelines), which may limit generalizability to younger or less active populations.

**3. d) Response from authors:** We agree with the noted limitations of our sample, and have acknowledged this in our Discussion section and added detail for clarity with **additions in bold** (*clean Manuscript Page 27, Line 585; tracked changes Manuscript Page 27 Line 851):*

*“Another limitation is that roughly 70% of our international sample exceeded the WHO exercise guidelines, which is likely not representative of the general population. The sample was also relatively skewed toward older adults, potentially due to recruitment methods (e.g., radio broadcasts, news articles, Facebook, etc.) that are likely not as popular among younger audiences.* ***This may limit the generalizability of results to younger or less active populations,*** *although the fact remains that among a large population of adults with a tendency towards regular exercise, a positive association was observed with mental health.”*

Ultimately, while we acknowledge this limitation, we again believe it does not discount our main finding: a statistically significant relationship between regular activity and mental health within our studied population of generally active adults.

Reviewer #2: Dear Authors,

Thank you for this well-designed study.
The study design, evaluations, and analyses appear to have been executed with rigor and depth. The level of detail provided regarding the tests and measurements conducted is deemed adequate. To further enhance the quality of the manuscript, please refer to the recommendations outlined below:

- The objective statement, located at the end of the introduction section, should be refined to provide greater clarity and emphasize the significance and potential beneficial impacts of the study.

**Response from author:** Thank you for this suggestion. To emphasize the significance and potential beneficial impacts of our study, we have added additional details to the final paragraph of the Introduction with **changes in bold** (*clean Manuscript Page 3, Line 98; tracked changes Manuscript Page 3 Line 234):*

“*To address some of the outstanding questions in this field, we performed an international online study in a large sample of younger, middle-aged, and older adults,* ***with the objective of better characterizing the relationships between physical activity, video game playing, cognition, and mental health****.* ***To measure various aspects of lifestyle and brain health, along with demographics and other health-related information, we deployed multiple validated questionnaires alongside a comprehensive cognitive test battery. The Creyos online cognitive testing platform, which includes twelve tasks that collectively assess a broad range of cognitive abilities, has been used in numerous large cohort studies (46–48) and provides detailed information about a variety of brain functions and the cognitive domains that they support. Based on the existing literature, we hypothesized that both exercise and video gaming would be related to cognition and/or mental health, although the exact nature of this relationship would likely vary in terms of the benefits observed – for example, the cognitive domains that showed associations with these lifestyle factors – and when during the lifespan they occur.*** *To our knowledge, simultaneously assessing the effect of physical activity and video gaming on both cognitive performance and mental health in such a large sample has not been done previously.* ***An advantage of this approach is that the profiles of brain health associated with each lifestyle factor can be directly compared because they are measured using the same instruments (i.e., questionnaires and cognitive tasks). The results from this largescale cross-sectional study will set the stage for future targeted studies of interventions and causal effects that will be of significant benefit for shaping health policy, informing individual lifestyle choices, and guiding future research on interventional approaches to brain health and longevity.***

- In the conclusions section, please include concise, point-by-point recommendations directed at scientists, outlining specific adjustments to enhance the quality of future research on this or related topics.

**Response from author:** We have added the following future directions to our conclusions section (*clean Manuscript Page 28, Line 632; tracked changes Manuscript Page 28 Line 899):*

“*To build upon these findings, future studies should aim to incorporate an objective assessment of physical activity level (e.g., wearable devices), as well as more comprehensive mental health assessment tools. Furthermore, a longitudinal study design would allow for more definitive and generalizable conclusions regarding the nature of the relationships between exercise, video gaming, and brain health.*”

Best regards

6. PLOS authors have the option to publish the peer review history of their article ([what does this mean?](https://track.editorialmanager.com/CL0/https:%2F%2Fjournals.plos.org%2Fplosone%2Fs%2Feditorial-and-peer-review-process%23loc-peer-review-history/1/010f0196e65e3044-7efc2bc1-d65e-4b80-9414-44b41640679a-000000/2VPk-kaYchkCxsc3y0xv3QjKOl_eKz86CzuAUxdtxCk=211)). If published, this will include your full peer review and any attached files.


If you choose “no”, your identity will remain anonymous but your review may still be made public.


**Do you want your identity to be public for this peer review?** For information about this choice, including consent withdrawal, please see our [Privacy Policy](https://track.editorialmanager.com/CL0/https:%2F%2Fwww.plos.org%2Fprivacy-policy/1/010f0196e65e3044-7efc2bc1-d65e-4b80-9414-44b41640679a-000000/rT8Kggzmz62ZbNDKoYtoLOjL7D8qsakH1p8fZ2VOVrs=211).

Reviewer #1: **Yes:** João Zambujal-Oliveira

Reviewer #2: No

[NOTE: If reviewer comments were submitted as an attachment file, they will be attached to this email and accessible via the submission site. Please log into your account, locate the manuscript record, and check for the action link "View Attachments". If this link does not appear, there are no attachment files.]

While revising your submission, please upload your figure files to the Preflight Analysis and Conversion Engine (PACE) digital diagnostic tool, [https://pacev2.apexcovantage.com/](https://track.editorialmanager.com/CL0/https:%2F%2Fpacev2.apexcovantage.com%2F/1/010f0196e65e3044-7efc2bc1-d65e-4b80-9414-44b41640679a-000000/YmbdnZ8DNv304Zd81HR2i5A79NU3a7SF7KRZZ_l7bQY=211). PACE helps ensure that figures meet PLOS requirements. To use PACE, you must first register as a user. Registration is free. Then, login and navigate to the UPLOAD tab, where you will find detailed instructions on how to use the tool. If you encounter any issues or have any questions when using PACE, please email PLOS at figures@plos.org. Please note that Supporting Information files do not need this step.
