# Supplementary material for: Characterizing the cognitive and mental health benefits of exercise and video game playing
Source: PLoS One. 2025 Oct 24;20(10):e0334924. doi: 10.1371/journal.pone.0334924 (PMC12551924; doi:10.1371/journal.pone.0334924)
Supplement: Attachment — Submitted filename: Response to Reviewers II.docx [file pone.0334924.s008.docx]

**Response to Reviewers II**

PONE-D-25-17797

Characterizing the Cognitive and Mental Health Benefits of Exercise and Video Game Playing

PLOS ONE

Dr. Mohammad Mofatteh, PhD, MPH, MSc, PGCert, BSc (Hons), MB BCh (c)
Academic Editor, PLOS ONE

Dear Dr. Mofatteh,

Thank you very much for relaying the Reviewers’ thorough assessment of our initial revision and response.

While Reviewer #2 is pleased with the manuscript in its current form, Reviewer #1 has raised a handful of additional points for further consideration. In our letter, we have attempted to address these issues by either making appropriate changes to the manuscript, or by directing this Reviewer to existing discussion points that already address their concern.

Thank you again for reconsidering our manuscript. Should anything remain unclear or require further attention, please do not hesitate to contact us.

Sincerely,

Sydni Paleczny ([spaleczn@uwo.ca](mailto:spaleczn@uwo.ca))

Conor Wild ([cwild@uwo.ca](mailto:cwild@uwo.ca))

on behalf of all co-authors.

Journal Requirements:

If the reviewer comments include a recommendation to cite specific previously published works, please review and evaluate these publications to determine whether they are relevant and should be cited. There is no requirement to cite these works unless the editor has indicated otherwise.

Additional Editor Comments:

Please provide a detailed point-by-point response along with updated version of the manuscript.


[Note: HTML markup is below. Please do not edit.]

Reviewers' comments:

Reviewer's Responses to Questions

**Comments to the Author**

1. If the authors have adequately addressed your comments raised in a previous round of review and you feel that this manuscript is now acceptable for publication, you may indicate that here to bypass the “Comments to the Author” section, enter your conflict of interest statement in the “Confidential to Editor” section, and submit your "Accept" recommendation.

Reviewer #1: (No Response)

Reviewer #2: All comments have been addressed

2. Is the manuscript technically sound, and do the data support the conclusions?

The manuscript must describe a technically sound piece of scientific research with data that supports the conclusions. Experiments must have been conducted rigorously, with appropriate controls, replication, and sample sizes. The conclusions must be drawn appropriately based on the data presented.

Reviewer #1: Partly

Reviewer #2: Yes

3. Has the statistical analysis been performed appropriately and rigorously?

Reviewer #1: No

Reviewer #2: Yes

4. Have the authors made all data underlying the findings in their manuscript fully available?

The [PLOS Data policy](http://track.editorialmanager.com/CL0/http:%2F%2Fwww.plosone.org%2Fstatic%2Fpolicies.action%23sharing/1/010f01981804972c-1297276e-2b8e-420c-b5f2-0222c77c0b8a-000000/0dsL-q1eRgHvRlly3wenhlU5er4cVgvnxMovKmAPssE=220) requires authors to make all data underlying the findings described in their manuscript fully available without restriction, with rare exception (please refer to the Data Availability Statement in the manuscript PDF file). The data should be provided as part of the manuscript or its supporting information, or deposited to a public repository. For example, in addition to summary statistics, the data points behind means, medians and variance measures should be available. If there are restrictions on publicly sharing data—e.g. participant privacy or use of data from a third party—those must be specified.

Reviewer #1: Yes

Reviewer #2: Yes

5. Is the manuscript presented in an intelligible fashion and written in standard English?

PLOS ONE does not copyedit accepted manuscripts, so the language in submitted articles must be clear, correct, and unambiguous. Any typographical or grammatical errors should be corrected at revision, so please note any specific errors here.

Reviewer #1: Yes

Reviewer #2: Yes

6. Review Comments to the Author

Please use the space provided to explain your answers to the questions above. You may also include additional comments for the author, including concerns about dual publication, research ethics, or publication ethics. (Please upload your review as an attachment if it exceeds 20,000 characters)

Reviewer #1:

- Provide justification for why Creyos is suitable for all age groups.

- **Response from authors:** We have added the following information (additions in **bold**) to Page 8 Lines 6-9:
  - “The 12-task Creyos battery assesses aspects of memory, attention, planning, and reasoning, using a web-based interface that can be self-administered at home through any internet browser [(46)](https://www.zotero.org/google-docs/?daduj8). The Creyos battery has been used in similar large-scale studies to assess the effects of sleep [(47)](https://www.zotero.org/google-docs/?vfJQf3), COVID-19 [(48)](https://www.zotero.org/google-docs/?wmEhXs), and lifestyle factors on cognitive functioning among the general population [(54)](https://www.zotero.org/google-docs/?o4WpU3) **across the lifespan (55) in studies with children and adolescents (56,57) and older adults (54,58). Importantly, several studies have demonstrated that older adults with limited experience with technology can manage digital cognitive assessments, producing data that is as reliable as in-person assessment (59-61).**

- Define "cognitive domains" with concrete examples.

- **Response from authors:** We have added the following information (additions in **bold**) to Pages 8 Line 23:
  - “These included three measures derived from a factor analysis [(46,47)](https://www.zotero.org/google-docs/?EWSdkx) that reflect performance within three broad cognitive domains: 1) short-term memory (STM; **the ability focus on and maintain task-relevant information in short-term memory)**, 2) reasoning **(the ability to mentally transform information according to logical rules)**, and 3) verbal performance **(the processing of information within the verbal or linguistic domain). These interpretations are derived from the characteristics shared by the tasks that load most heavily on each of the three factors observed by Hampshire et al. (2012), and are supported by neuroimaging results showing that distinct (and functionally consistent) brain networks are recruited by these combinations of tasks.”**

- Acknowledge the limitations of self-reporting, which are noted in both the physical activity and gaming questionnaires; suggested solutions (e.g., categorical classification) should be clearly presented.

- **Response from authors:** The reviewer has requested the following changes:

(1) That we acknowledge the limitations of self-reporting

(2) That we suggest solutions (e.g., categorical classification)

We have already acknowledged the first point on Page 26 Line 16:

“We also must acknowledge the limitations of using self-reported estimates of the frequency and duration of physical exercise and playing video games as it is well known that people over-report [(51,108)](https://www.zotero.org/google-docs/?1qRGLw).”

And, we offer *three* solutions to this problem: 1) categorization (our solution, given the data available), 2) accelerometers (not feasible for this kind of study), and 3) personal fitness trackers (which have their own issues that must be addressed in future work). These points are are all presented on Page 26 Line 19:

- “To overcome this, we categorized individuals based on their self-reported frequency of the activity; although the accuracy of individuals’ self-report judgements may be low, the classification of individuals into categories (i.e., high or low extremes) based on their estimates is substantially better [(37,52,53)](https://www.zotero.org/google-docs/?F5JWmP). Obviously, objective data would produce more accurate measurements, even for classifying individuals [(109)](https://www.zotero.org/google-docs/?lgr4F3)[,](https://www.zotero.org/google-docs/?TUpYCN) but within the present study obtaining gold standard accelerometer data was simply not feasible due to the international online design and costs associated with our large sample size. Personal activity monitors (e.g., FitBit, Apple Watch, etc.) provide an interesting potential alternative for acquiring objective physical activity data, but there are significant issues that first need to be overcome, such as addressing concerns related to data privacy, establishing the quality and reliability of data collected from dozens of different device types across multiple platforms with proprietary measurements, and the need for techniques to harmonize data from across these platforms [(109)](https://www.zotero.org/google-docs/?SuDhHi). Future work will investigate the feasibility of incorporating participants’ existing personal physical activity tracking devices into an online study of this type.”

- Since recruitment was conducted online, specify what measures were taken—or could be taken—to mitigate bias toward individuals with higher internet literacy or an interest in cognition-related topics.

- **Response from authors:** Indeed, bias may have affected the general representativeness of our sample, which we have mentioned in the Discussion (additions in **bold**) on Page 27 Line 19:
  - “Another limitation is **demographic bias. For example,** our international sample **was relatively skewed toward older adults, and roughly 70% of the sample** exceeded the WHO exercise guidelines. **This may** potentially be due to recruitment methods (e.g., radio broadcasts, news articles, Facebook, etc.) that are likely not as popular among younger audiences. **In any case, the sample is likely not representative of the general population** which may limit the generalizability of results to younger or less active populations”

- The filtering criterion “Speaks >25 languages” requires a stronger justification.

- **Response from authors:** Thank you for pointing this out. The correct filtering criterion should read “Speaks >7 languages” which was determined through visual inspection of histogram data to identify outliers. We have updated this criterion in Table 1, and added the following information (additions in **bold**) to Page 11 Lines 9-10:
  - “Those who reported unusually high numbers for numeric entry questions **(according to visual inspection of histogram data to identify outliers)**, who had a cognitive score more than 4 standard deviations from the mean, or who were missing any data from the cognitive tests, mental health screeners, the PAAQ, or the VGQ were also excluded.”

- Some thresholds (e.g., “>20 units of alcohol/day”) seem arbitrary; provide a rationale or cite relevant literature.

- **Response from authors:** Thank you again for pointing this out. This threshold corresponds to the upper ~5% of the distribution and corresponds to discontinuities identified from visual inspection of histogram data. We have added detail (additions in **bold**) to our filtering criteria on Page 11 Lines 9-10**:**
  - “Those who reported unusually high numbers for numeric entry questions **(according to visual inspection of histogram data to identify outliers)**, who had a cognitive score more than 4 standard deviations from the mean, or who were missing any data from the cognitive tests, mental health screeners, the PAAQ, or the VGQ were also excluded.”

- Consider removing repetition between text and tables (e.g., mental health results) to improve conciseness.

- **Response from authors:** Thank you for this suggestion. We have removed the aspects of the text that repeat data in tables for conciseness (Page 16).

- Clarify how demographic bias may have led to an overrepresentation of active and older adults.

- **Response from authors:** We have reworded and added detail (additions in **bold**) to Page 27 Line 19:
  - “Another limitation **is demographic bias. For example,** our international sample **was relatively skewed toward older adults, and roughly 70% of the sample** exceeded the WHO exercise guidelines. This may potentially be due to recruitment methods (e.g., radio broadcasts, news articles, Facebook, etc.) that are likely not as popular among younger audiences. **In any case, the sample is likely not representative of the general population,** which may limit the generalizability of results to younger or less active populations.”

- Address any potential mismatch in measurement timing, such as physical activity being assessed weekly while gaming is assessed annually.

- **Response from authors:** We have addressed the mismatch in measurement timing on Page 27 Line 7, and reworded for clarity (additions in **bold**):
  - “It is also worth noting the different time frames evaluated by the questionnaires for physical activity (PAAQ: hours of activity **per week** **over** the past week) and video gaming (VGQ; average hours **of video gaming** per week over the **p**ast year). This was simply a consequence of using established questionnaires, though we considered this discrepancy by asking participants if their PA estimate was representative of their usual routine, **which most participants indicated was the case.** **Nonetheless, it could be that any cognitive benefits afforded by long-term physical exercise were not detected using this particular assessment; however, this seems unlikely given previous research that has demonstrated a positive relationship between acute exercise and cognition (12).**

- Explain why the observational nature of the data limits conclusions about directionality.

- **Response from authors:** We have acknowledged that our study design does not allow us to draw any conclusions surrounding causality, and added the following detail (additions in **bold**) to Page 28 Line 9:
  - “While we observed significant associations between physical exercise and mental health and between video gaming and cognitive performance, it is not possible to say that this is a causative relationship based on the current data. For example, more depressed or more anxious individuals may be less likely to exercise than their less depressed or less anxious counterparts. This explanation seems unlikely, however, because the association between physical activity and mental health scores was weakest among those with higher depression or anxiety ratings, suggesting that these symptoms were not the main drivers of the performance differences. Where video gaming and cognition is concerned, the direction of the effect seems rather less open to question. That is to say, while it is theoretically possible that those who have better cognition are more likely to play video games regularly, it seems more probable that regular gaming exerts a direct effect on aspects of cognitive performance. In any case, the cross-sectional design of the present study does not allow us to draw any definitive conclusions surrounding causality **or directionality**. Future research may benefit from longitudinal follow-up to further investigate and determine the nature of this relationship over time.”

- Suggest directions for future research, such as longitudinal study designs.

- **Response from authors:** Please see Page 29 Line 16 (relevant text highlighted):
  - “To build upon these findings, future studies should aim to incorporate an objective assessment of physical activity level (e.g., wearable devices), as well as more comprehensive mental health assessment tools. Furthermore, a longitudinal study design would allow for more definitive and generalizable conclusions regarding the nature of the relationships between exercise, video gaming, and brain health. Research in this domain is important to identify which aspects of brain health are affected by certain lifestyle factors, in order to support health policies and inform individuals to choose activities that will preserve their mental and cognitive health with age.”

Reviewer #2: Dear Authors,

It seems that the suggested revisions were made successfully. With its current form, I believe that this manuscript can be a valuable addition to practical sport science applications.

Best regards,

7. PLOS authors have the option to publish the peer review history of their article ([what does this mean?](https://track.editorialmanager.com/CL0/https:%2F%2Fjournals.plos.org%2Fplosone%2Fs%2Feditorial-and-peer-review-process%23loc-peer-review-history/1/010f01981804972c-1297276e-2b8e-420c-b5f2-0222c77c0b8a-000000/jeQN0I_VO_I9Tm9EPV8JYJAcOqPJjx5XFrBoHzX8RBc=220)). If published, this will include your full peer review and any attached files.


If you choose “no”, your identity will remain anonymous but your review may still be made public.


Do you want your identity to be public for this peer review? For information about this choice, including consent withdrawal, please see our [Privacy Policy](https://track.editorialmanager.com/CL0/https:%2F%2Fwww.plos.org%2Fprivacy-policy/1/010f01981804972c-1297276e-2b8e-420c-b5f2-0222c77c0b8a-000000/fnKsp4sb7VEhGoVBSeYdRKpqAbO31rWm2KkU5dHuKW0=220).

Reviewer #1: Yes: João Zambujal-Oliveira

Reviewer #2: No
